# Postpartum Uterine Diseases in Dairy Cattle: Integrating Microbiology, Immunology, and Reproductive Physiology

**DOI:** 10.3390/microorganisms14081645

**Published:** 2026-07-28

**Authors:** Ramanathan Kasimanickam, Priunka Bhowmik, Zhihua Jiang

**Affiliations:** 1 College of Veterinary Medicine, Washington State University, Pullman, WA 99164, USA; 2 College of Agricultural, Human, and Natural Resource Sciences, Washington State University, Pullman, WA 99163, USA; priunka.bhowmik@wsu.edu (P.B.); jiangz@wsu.edu (Z.J.)

**Keywords:** cattle, uterine diseases, uterine microbiota, microbial dysbiosis, immune regulation, virulence factors

## Abstract

Postpartum uterine diseases are among the most prevalent and economically important reproductive disorders affecting dairy cattle worldwide. These conditions, including metritis, clinical and subclinical endometritis, and pyometra, develop during the postpartum transition period when physiological, metabolic, endocrine, and immunological adaptations increase susceptibility to microbial invasion and persistent uterine inflammation. Although bacterial contamination of the postpartum uterus is nearly universal, healthy cows generally restore uterine homeostasis through coordinated immune responses, microbial regulation, and effective uterine involution. Failure of these defense mechanisms results in microbial dysbiosis, impaired endometrial repair, reduced fertility, and substantial economic loss. Major pathogens associated with postpartum uterine disease include *Escherichia coli*, *Trueperella pyogenes*, *Fusobacterium necrophorum*, *Prevotella* spp., and other anaerobic bacteria that interact synergistically to promote inflammation, tissue damage, and reproductive dysfunction. Advances in next-generation sequencing, metagenomics, and metatranscriptomics have transformed understanding of the postpartum uterine microbiota and host–microbe interactions involved in disease pathogenesis. This review synthesizes current evidence regarding uterine physiology, microbial ecology, immune regulation, virulence mechanisms, dysbiosis, diagnostic approaches, and emerging omics-based technologies relevant to postpartum uterine disease in dairy cattle. Particular emphasis is placed on the ecological and physiological interactions linking microbial succession, endocrine recovery, metabolic stress, and immune competence during the postpartum period. The review further discusses translational opportunities for precision diagnostics, microbiome-informed interventions, antimicrobial stewardship, and integrated herd management strategies to improve reproductive efficiency, animal welfare, and dairy herd sustainability.

## 1. Introduction

The transition period is one of the most critical phases in a dairy cow’s life, marked by major physiological, metabolic, microbial, and immunological changes [1,2]. During this time, the uterus undergoes involution, returning to its pre-pregnancy state [3,4]. Involution involves expelling the fetal membranes, remodeling tissues, and clearing the postpartum lochia, which contains mucus, blood, cellular debris, and microbes. Bacterial contamination is nearly universal during the first two weeks after parturition, but most healthy cows regulate microbial colonization and restore uterine balance [5]. Evidence suggests the postpartum uterus, even in healthy cows, is not always sterile but instead supports a dynamic microbial community that may aid uterine physiology and immune regulation. Disrupting microbial balance or delaying uterine clearance can cause postpartum diseases such as metritis, endometritis, and pyometra, thereby impairing reproduction, animal welfare, and farm profitability [6,7].

Postpartum uterine diseases are significant health issues in dairy cattle, ranking with mastitis and lameness due to their high prevalence and economic impact [6,7]. These diseases range from localized inflammation to severe systemic illness. Metritis is an acute disease usually seen within 10 days after calving, characterized by an enlarged, atonic uterus and foul-smelling, watery discharge [6]. Affected cows often have systemic signs such as fever, anorexia, and depression. In contrast, endometritis develops later and mainly affects the endometrial lining. Clinical endometritis presents as mucopurulent uterine discharge, whereas subclinical endometritis is diagnosed by increased neutrophils on endometrial cytology [8,9,10,11,12]. Pyometra is less common, marked by pus accumulation in the uterus, a persistent functional corpus luteum (CL), and absence of estrus, eventually leading to infertility [6].

The cause of postpartum uterine disease is multifactorial, but bacterial infection is the main trigger [13,14,15,16,17]. Opportunistic pathogens enter primarily during calving, when barriers are disrupted. The main pathogens include *Escherichia coli*, *Trueperella pyogenes*, *Fusobacterium necrophorum*, and anaerobic bacteria such as *Prevotella* spp. These microbes use diverse virulence factors to adhere to the uterine lining, evade immune defenses, and damage tissue. Disease progression depends on microbial interactions, as colonization by one organism can increase the pathogenicity of another. For example, early colonization by *E. coli* can promote later infection by *T. pyogenes*, heightening inflammation and delaying endometrial repair. Emerging evidence suggests other microbes, such as *Mycoplasma* and fungi, may also contribute to postpartum uterine dysbiosis and inflammation, though their roles remain unclear.

Understanding the uterine microbiota is key to learning how these disorders develop. Traditional culture techniques gave useful information about pathogens but missed much of the microbial diversity in the uterus. Modern molecular methods, especially next-generation sequencing (NGS), provide a deeper view of the uterine microbiome [18,19,20]. These studies have found a broader range of microbial taxa in both healthy and diseased uteri, including phyla like Firmicutes, Bacteroidetes, Proteobacteria, and Fusobacteria. Changes in the microbial community, rather than just a pathogen’s presence, have been linked to disease. Thus, recent research has focused on host–microbiome interactions and on how microbial dysbiosis leads to persistent inflammation, impaired tissue repair, and reproductive dysfunction.

In parallel with microbial factors, the maternal immune response plays a pivotal role in determining postpartum uterine contamination outcomes. The bovine innate immune system is crucial for recognizing and responding to microbial invasion. Endometrial cells express pattern recognition receptors, including Toll-like receptors (TLRs), which detect pathogen-associated molecular patterns (PAMPs) such as lipopolysaccharide (LPS) from Gram-negative bacteria [21,22]. Activating these receptors triggers immune responses, including the production of pro-inflammatory cytokines (IL-1β, TNF-α, IL-6), the recruitment of leukocytes via chemokine signaling, and the activation of neutrophils and macrophages [21,22]. However, postpartum immune competence is often compromised by calving stress, negative energy balance, and endocrine changes, including peripartum hormonal fluctuations and elevated progesterone [23]. As a result, impaired immune function can slow pathogen clearance, promoting persistent infection, chronic inflammation, and endometrial damage.

Postpartum uterine diseases have large economic effects. Affected cows often conceive later, need more inseminations, and remain open longer [6,11]. This leads to more culling, higher veterinary costs, and lower productivity.

Collectively, the postpartum uterus represents a highly dynamic ecosystem shaped by interactions among microbial populations, host immune responses, endocrine regulation, and metabolic status. Consequently, effective prevention and management of uterine disease require a multidisciplinary approach integrating microbiology, immunology, endocrinology, and herd management practices. Recent advances in culture-independent sequencing and other high-throughput technologies have substantially improved understanding of host–microbe interactions and are expected to facilitate more precise diagnostic and therapeutic strategies.

Disease progression is not determined solely by microbial presence. Rather, postpartum uterine disease represents a systems-level disorder arising from dynamic interactions among four interconnected biological processes: (1) microbial succession, (2) restoration of immune competence, (3) endocrine and metabolic adaptation, and (4) endometrial repair during uterine involution. Disturbance of any component influences the others through reciprocal feedback mechanisms. For example, negative energy balance and endocrine disruption impair neutrophil function and pathogen clearance, whereas persistent dysbiosis amplifies inflammatory signaling, delays uterine repair, and postpones resumption of ovarian cyclicity. Understanding these interconnected processes provides the conceptual framework for this review.

This review synthesizes current knowledge regarding postpartum uterine diseases by integrating microbiology, immunology, reproductive physiology, endocrine regulation, and transition cow biology. Particular emphasis is placed on host–microbe interactions, microbial dysbiosis, disease pathogenesis, emerging diagnostic approaches, and evidence-based prevention and management strategies while highlighting important knowledge gaps and future research priorities.

## 2. The Bovine Uterus

The bovine uterus is bicornuate and consists of three distinct layers: the outer perimetrium, the middle myometrium, and the inner endometrium [24]. The myometrium is composed of circular, longitudinal, and vascular muscle layers, whereas the endometrium contains both mucosal and submucosal components. In healthy cows, the uterus is generally considered sterile between pregnancies, although recent evidence suggests that low-abundance resident microbial populations may be present under physiological conditions. Anatomical structures, including the vulva, vestibule, cervix, and associated muscular sphincters, provide important physical barriers that limit the spread of ascending microbial contamination [24]. The uterine environment is predominantly anaerobic, and uterine pH typically ranges from 6.8 to 8.3 depending on diet and stage of the estrous cycle [25,26].

During pregnancy, the endometrium develops specialized structures known as caruncles, highly vascularized fibrous protrusions that contain deep villi and folds. These maternal structures interact with the fetal cotyledons to form placentomes, the functional sites of nutrient and gas exchange between maternal and fetal circulations [27,28,29]. Following calving, the uterus undergoes involution, a process involving progressive reduction in uterine size and restoration of reproductive tract integrity. Although substantial involution occurs within approximately three weeks postpartum, complete uterine recovery may require 47–50 days. Caruncular regression typically occurs within two weeks, while the decidual layer is shed approximately 5–8 days after parturition. During this period, lochia, a uterine discharge composed of blood, shed decidual tissue, inflammatory exudate, and residual fetal membranes, is expelled through myometrial contractions and gradually changes in color and consistency over 2–3 weeks [30,31,32]. Because lochia contains abundant organic material, it can serve as a favorable substrate for microbial proliferation during the postpartum period.

Bacterial contamination of the uterus is nearly universal during the first two weeks after calving [4]. Nevertheless, healthy cows typically eliminate microbial contaminants, regenerate the endometrial lining, and resume normal ovarian cyclicity during uterine involution [33,34]. Failure of these recovery processes may predispose cows to postpartum uterine diseases, including metritis, endometritis, and pyometra.

While uterine anatomy and involution are well established, increasing evidence indicates that structural recovery alone does not predict restoration of uterine health. Recent microbiome studies suggest that successful involution should be considered together with microbial succession and immune resolution, emphasizing that anatomical recovery and functional recovery are not necessarily synonymous.

## 3. The Endocrine System

Restoration of ovarian cyclicity during the postpartum period is regulated by coordinated endocrine signaling involving the hypothalamic–pituitary–ovarian axis. Gonadotropin-releasing hormone secreted from the hypothalamus stimulates the release of follicle-stimulating hormone and luteinizing hormone (LH) from the anterior pituitary gland [35,36]. These gonadotropins promote follicular growth and maturation, leading to increased estrogen production, particularly estradiol (E2), which is essential for ovulation and estrous behavior. Progesterone produced by the CL subsequently regulates endometrial function and suppresses premature ovulation by modulating hypothalamic and pituitary activity [35,36].

Approximately 17–21 days after ovulation, prostaglandin F2α released by the endometrium induces luteolysis, leading to regression of the CL and a decline in progesterone concentrations. This hormonal shift permits the development of a new dominant follicle, increased E2 secretion, and initiation of a subsequent estrous cycle [35,36]. Disruption of these endocrine processes during the postpartum period can impair ovarian cyclicity and reduce fertility in dairy cows.

Although endocrine regulation of postpartum cyclicity has been extensively characterized, the mechanisms through which endocrine signals influence microbial community composition remain poorly understood. Future investigations integrating endocrine profiles with longitudinal microbiome analyses may clarify bidirectional host–microbe interactions during postpartum recovery.

## 4. Underlying Mechanisms of Uterine Disease

The pathogenesis of postpartum uterine disease involves complex interactions among pathogenic microorganisms, host immune responses, endocrine regulation, and tissue repair mechanisms [13,14,15,16,37]. Following parturition, opportunistic pathogens such as *T. pyogenes*, *E. coli*, and other anaerobic bacteria may colonize the uterus and initiate inflammatory responses within the endometrium [12,13,14,15,16,17,18,19,20,21,22,23,38,39,40]. Recognition of microbial components by the uterine immune system stimulates the production of pro-inflammatory cytokines, chemokines, and other inflammatory mediators to eliminate invading pathogens. Although these responses are essential for host defense, excessive or persistent inflammation can negatively affect reproductive function.

One major consequence of uterine inflammation is disruption of hypothalamic–pituitary–ovarian signaling, particularly suppression of LH secretion from the pituitary gland [41]. Because LH is essential for follicular maturation and ovulation, inflammatory cytokines released during uterine infection can interfere with normal ovarian function and delay resumption of cyclicity [42,43,44].

Consequently, affected cows frequently experience prolonged postpartum anestrus and reduced fertility. Inflammation associated with uterine infection may also impair uterine involution and endometrial repair. Under normal physiological conditions, the uterus progressively returns to its pre-pregnancy state within several weeks postpartum. However, persistent infection prolongs tissue remodeling and delays restoration of a functional uterine environment [34,45]. An incompletely involuted uterus is less capable of supporting embryo implantation and early embryonic development, thereby reducing conception success in subsequent breeding cycles. Furthermore, inflammatory alterations in the uterine microenvironment, including oxidative stress, altered cytokine profiles, and tissue damage, may directly compromise embryo survival and implantation.

## 5. The Immune System

Postpartum dairy cows are particularly susceptible to uterine infections because parturition disrupts normal physical barriers and facilitates bacterial entry into the reproductive tract. Disease develops when microbial contamination exceeds the capacity of uterine defense mechanisms, potentially resulting in puerperal metritis, clinical or subclinical endometritis, and pyometra. The innate immune system constitutes the primary defense against invading pathogens. Endometrial epithelial and stromal cells express pattern recognition receptors, including TLRs, which recognize PAMPs derived from microorganisms [46]. Specifically, TLR1, TLR2, and TLR6 recognize bacterial lipoproteins, TLR3, TLR7, TLR8, and TLR9 detect viral nucleic acids, and TLR4 recognizes LPS from Gram-negative bacteria [47]. Additional protection is provided by antimicrobial peptides such as defensins and by acute-phase proteins that contribute to innate immune defense [47].

Activation of TLR signaling pathways stimulates production of pro-inflammatory cytokines and chemokines, including IL-1, IL-6, and TNF-α, thereby initiating local inflammatory responses [48,49,50,51]. These mediators recruit neutrophils to the site of infection, where they adhere to, phagocytize, and destroy invading microorganisms. Other immune cells, including macrophages, eosinophils, and additional leukocyte populations, subsequently contribute to pathogen clearance and tissue repair. In more persistent or severe infections, activation of the adaptive immune system may occur through recruitment of lymphocyte populations, including B cells and T cells [49,50,51,52].

Despite these protective mechanisms, immune competence during the postpartum period is often compromised by calving-associated stress and the metabolic demands of lactation [53,54]. Nutritional status, negative energy balance, and excessive fat mobilization further influence immune function and inflammatory responses [53,54]. Moreover, sustained production of pro-inflammatory cytokines such as TNF-α and IL-6 may exacerbate tissue damage and contribute to chronic uterine inflammation [55]. Endocrine factors also modulate postpartum immunity; for example, progesterone exerts immunomodulatory and immunosuppressive effects that are essential for maintenance of pregnancy but may impair antimicrobial defense mechanisms during the postpartum transition period.

Most available studies evaluate individual cytokines or immune cell populations independently. However, uterine immunity functions as a coordinated network influenced by metabolic status, endocrine signaling, and microbial ecology. Future studies should integrate these pathways using systems biology approaches rather than isolated immune markers.

## 6. Types of Postpartum Uterine Diseases

Postpartum uterine diseases, including the retained placenta–metritis complex, endometritis, and pyometra, represent major health concerns in dairy cattle because of their detrimental effects on reproductive performance, animal welfare, and farm profitability. These disorders typically arise from bacterial contamination of the uterus during or shortly after calving. Diagnosis is based on evaluation of uterine discharge, assessment of systemic clinical signs, and endometrial cytology.

Endometritis is classified as either clinical or subclinical. Clinical endometritis is characterized by inflammation of the endometrial lining accompanied by purulent or mucopurulent uterine discharge occurring ≥28 days postpartum in the absence of systemic illness [6,8,9]. In contrast, subclinical endometritis lacks visible uterine discharge and is diagnosed based on elevated neutrophil counts in endometrial cytology samples, typically >18% between 21 and 33 days postpartum or >10% between 34 and 47 days postpartum [11,12]. While the broader clinical thresholds (>1% to >18% polymorphonuclear neutrophils (PMNs) depending on the timeframe) are used to capture more cases in the field, that >6% PMN from 28 days in milk onwards has been used as a threshold because it provides a more stringent and consistent baseline. Subclinical endometritis affects approximately 30–50% of dairy cows, whereas clinical endometritis occurs in approximately 5–25% of cows.

Metritis is a more severe postpartum uterine disorder involving inflammation of all uterine layers, extending from the endometrium to the serosa. It is characterized by an enlarged, atonic uterus and foul-smelling, watery uterine discharge. Cows affected with metritis frequently exhibit systemic clinical signs, including fever, anorexia, weakness, reduced milk production, tachycardia, and depression [15,38]. The prevalence of metritis ranges from 5 to 20% in dairy cows and is considered particularly important because of its potential progression to septic-toxic metritis, a life-threatening condition [15,38].

Pyometra is characterized by the accumulation of purulent material within the uterine lumen in the presence of a persistent functional corpus luteum, resulting in suppression of estrous cyclicity [39]. Affected cows generally do not exhibit systemic illness but commonly present with anestrus and infertility. Pyometra accounts for less than 5% of clinical uterine disease cases in dairy cattle [40]. Although pyometra occurs less frequently than metritis or endometritis, it remains an important cause of prolonged infertility in dairy cattle. The disease typically develops following persistent bacterial infection in conjunction with maintenance of a functional corpus luteum, resulting in cervical closure and accumulation of purulent uterine contents. *T. pyogenes*, anaerobic bacteria including *F. necrophorum* and *Prevotella* spp., and mixed bacterial infections are commonly isolated from affected uteri. Unlike acute metritis, systemic illness is generally absent because infection remains localized within the uterine lumen. Diagnosis relies on rectal palpation, ultrasonography demonstration of intrauterine echogenic fluid, and identification of a persistent corpus luteum. Treatment primarily involves administering prostaglandin F_2_α to induce luteolysis and cervical relaxation, facilitating uterine drainage, with antimicrobial therapy reserved for selected cases based on clinical assessment and antimicrobial stewardship principles. Early diagnosis improves reproductive prognosis and reduces prolonged infertility.

## 7. Diagnosis of Postpartum Uterine Diseases

Accurate diagnosis of postpartum uterine diseases is essential for timely intervention and improved reproductive outcomes in dairy cattle. Diagnostic approaches typically involve evaluation of clinical signs, vaginal discharge characteristics, rectal palpation, ultrasonography, and cytological assessment of the endometrium. Because postpartum bacterial contamination is common in healthy cows, distinguishing physiological uterine involution from pathological infection remains a major clinical challenge [55,56,57,58,59].

Clinical examination frequently includes assessment of vaginal discharge using vaginoscopy or gloved-hand evaluation. Fetid, watery, reddish-brown discharge is commonly associated with metritis, whereas purulent or mucopurulent discharge observed ≥28 days postpartum is indicative of clinical endometritis [6,8,9]. Rectal palpation and transrectal ultrasonography are also widely used to evaluate uterine size, fluid accumulation, endometrial thickness, and ovarian structures. Ultrasonography provides additional diagnostic value by allowing visualization of intrauterine fluid, echogenic debris, and a persistent CL associated with pyometra [11,60].

Subclinical endometritis cannot be diagnosed through external clinical signs and therefore requires cytological evaluation of the endometrium. Endometrial cytology is commonly performed using uterine lavage, cytobrush, or biopsy techniques to quantify PMN percentages [11,12]. Elevated PMN proportions are indicative of persistent uterine inflammation and are associated with reduced fertility. Cytological diagnosis is considered one of the most reliable methods for identifying subclinical uterine disease because it enables the detection of inflammation before overt clinical manifestations become apparent [11,12].

Microbiological culture techniques have traditionally been used to identify uterine pathogens; however, these approaches are limited by the inability to culture many anaerobic or fastidious microorganisms. Consequently, molecular approaches such as polymerase chain reaction (PCR), quantitative PCR (qPCR), and NGS have increasingly been employed to characterize the postpartum uterine microbiome and identify microbial dysbiosis associated with disease progression [18,19,20]. These technologies have expanded understanding of host–microbe interactions and may contribute to the development of more precise diagnostic biomarkers for postpartum uterine diseases.

In addition to microbial assessment, inflammatory biomarkers have emerged as potential diagnostic tools for evaluating uterine health. Increased concentrations of acute-phase proteins, cytokines, and metabolic indicators have been associated with postpartum uterine inflammation and impaired reproductive performance [61,62,63]. Integration of clinical, cytological, microbiological, and molecular diagnostic approaches may therefore improve early disease detection, facilitate targeted therapeutic interventions, and enhance reproductive management in dairy herds.

Current diagnostic approaches primarily identify established disease rather than predicting disease progression. Integration of microbiome profiling, inflammatory biomarkers, and metabolic indicators may improve early identification of cows at greatest risk before clinical disease becomes apparent.

## 8. Risk Factors and Predisposing Conditions

The development of postpartum uterine diseases in dairy cattle is influenced by numerous interrelated risk factors involving nutrition, metabolism, calving-associated trauma, immune competence, and herd management practices. These factors compromise uterine defense mechanisms, delay involution, and increase susceptibility to microbial colonization and persistent inflammation.

Retained placenta is considered one of the most important predisposing factors for postpartum uterine disease because retained fetal membranes provide an ideal substrate for bacterial proliferation and impair normal uterine clearance mechanisms [56,57,58,63,64]. Dystocia, twin births, stillbirth, and prolonged calving are also strongly associated with increased risk of metritis and endometritis due to excessive tissue trauma, contamination, and delayed uterine recovery [57,65,66]. Furthermore, cesarean section and obstetrical manipulations may increase bacterial introduction into the reproductive tract and exacerbate uterine inflammation.

Metabolic and nutritional disorders during the transition period also play critical roles in disease susceptibility. Negative energy balance, excessive fat mobilization, ketosis, and hypocalcemia impair immune function and reduce the ability of postpartum cows to eliminate uterine pathogens effectively [64]. Deficiencies in trace minerals and vitamins, including selenium, zinc, and vitamin E, have likewise been associated with compromised neutrophil activity and increased incidence of uterine disease [64,65]. Because high-producing dairy cows experience substantial metabolic stress during early lactation, they are particularly vulnerable to postpartum immune dysregulation and reproductive disorders [66].

Immune suppression during the periparturient period further contributes to disease pathogenesis. Physiological stress associated with calving and initiation of lactation alters leukocyte function, cytokine production, and inflammatory regulation [61,63]. Reduced neutrophil migration, impaired phagocytic capacity, and altered endocrine signaling collectively decrease uterine immune competence, thereby facilitating persistence of pathogenic microorganisms within the postpartum uterus.

Environmental and management-related factors additionally influence the prevalence of postpartum uterine diseases [67]. Poor hygiene during calving, overcrowding, heat stress, inadequate nutrition, and improper transition cow management can increase exposure to pathogens and impair postpartum recovery [68,69]. Herd-level factors such as parity, season, housing conditions, and reproductive management practices have also been associated with variation in disease incidence. Multiparous cows, for example, are generally more susceptible to uterine disease due to increased metabolic demands and cumulative reproductive stress [70].

Collectively, postpartum uterine diseases arise from complex interactions among microbial exposure, host immunity, endocrine status, metabolic health, and environmental management. Understanding these interconnected risk factors is essential for developing effective preventive strategies to improve uterine health, reproductive efficiency, and overall dairy herd productivity.

## 9. Impact of Uterine Diseases

Postpartum uterine diseases significantly impair cow welfare by causing discomfort, pain, and systemic illness. Elevated concentrations of acute-phase proteins in the blood of affected cows suggest that uterine bacterial contamination contributes substantially to this inflammatory condition [71]. Clinical signs commonly observed in cows with endometritis and pyometra include reduced appetite, decreased milk production, and fever [71].

Uterine diseases also have a profound negative impact on reproductive performance. Cows with endometritis exhibit a 17–20% lower conception rate and require approximately 27% longer to become pregnant compared with unaffected herd mates [72]. Additionally, cows with metritis experience prolonged intervals from calving to conception, often extending to 122–124 days in milk (DIM), compared with approximately 95 DIM in healthy cows [29]. Even after treatment, cows with clinical endometritis frequently demonstrate reduced fertility. As a result, poor reproductive performance contributes to culling, increased veterinary intervention, and substantial economic losses in dairy production systems.

The fertility consequences of uterine disease are closely linked to disruptions in physiological, metabolic, and endocrine pathways. Negative energy balance and impaired immune function both contribute to the likelihood of disease development and persistence. These conditions are interrelated, as negative energy balance and compromised immunity can reduce the efficiency of uterine clearance mechanisms, thereby increasing susceptibility to postpartum uterine infections such as metritis, endometritis, and pyometra.

Beyond their effects on reproductive performance, postpartum uterine diseases impose considerable economic burdens on dairy farms. Economic losses arise through reduced milk production, increased veterinary costs, prolonged calving intervals, additional inseminations, and premature culling [73]. The primary expense associated with endometritis is the extended interval to conception, which can result in up to 56 additional open days [55]. Other significant costs include veterinary interventions, increased insemination frequency, and reduced milk yield. In the United States, the estimated annual cost per case of clinical endometritis is approximately $291, with national economic losses exceeding $650 million annually, assuming a 20% prevalence rate [73,74].

The economic burden of metritis is similarly substantial. In dairy cows, metritis is characterized by fetid, watery, red-brown vaginal discharge accompanied by systemic signs of illness, including fever, reduced feed intake, and decreased milk production [75]. Clinical postpartum metritis is estimated to occur in approximately 20% of dairy cows [76,77]. One economic analysis estimated that a single case of metritis resulted in approximately $340 in losses due to reduced milk production, impaired fertility, and treatment costs [76,77]. In a 1000-cow dairy herd, this could correspond to an annual economic loss of approximately $204,000. Another study estimated that the annual cost associated with metritis in U.S. dairy herds exceeded $665 million, with average losses of approximately $358 per affected cow [75,76,77]. These estimates emphasize the substantial economic consequences of postpartum uterine diseases and highlight the importance of effective prevention, early diagnosis, and management strategies to improve dairy herd health, productivity, and sustainability.

The preceding sections describe the anatomical, endocrine, and immunological environment in which postpartum disease develops. The following sections focus on microbial ecology, illustrating how alterations in microbial succession interact with host defense mechanisms to determine whether physiological uterine involution progresses toward recovery or persistent disease.

## 10. Microbiota Overview

The microbiota refers to the diverse community of microorganisms residing within a specific biological niche or environment [78]. Distinct microbial communities inhabit various body sites in both animals and humans. Because only a fraction of microorganisms can be cultured using conventional laboratory techniques, modern microbiome research increasingly relies on culture-independent approaches, particularly high-throughput sequencing technologies, to characterize microbial populations [79]. In dairy cows, most microbiome research has focused on the gastrointestinal tract; however, sequencing-based studies have also investigated the vaginal and hoof microbiota [80,81].

The rumen microbiota contributes substantially to host physiology by producing volatile fatty acids, vitamins, and microbial proteins, all of which are essential for animal health and productivity [82,83,84]. Dietary changes can influence ruminal pH, gas concentrations, volatile fatty acid production, and microbial composition, highlighting the dynamic relationship between the host environment and resident microbiota [85]. Similarly, alterations in the uterine environment during the postpartum period may influence microbial composition and function. Although the uterus was historically considered sterile outside pregnancy, recent evidence suggests that a transient uterine microbiota may exist and contribute to reproductive health or disease under certain physiological conditions [24,86].

## 11. Uterine Microbiota and Homeostasis

The uterine microbiota of dairy cows plays an important role in reproductive health and disease. Historically, the uterus was believed to remain sterile to support implantation and pregnancy maintenance. However, advances in microbiome research have challenged this concept [87,88,89]. Core bacterial phyla identified within the bovine uterine microbiota include Actinobacteria, Bacteroidetes, Firmicutes, Fusobacteria, Proteobacteria, and Tenericutes [88,89]. These microbial communities may help maintain a favorable uterine environment for implantation and fetal development. In uteri collected from Danish slaughterhouses, the predominant bacterial families included *Lachnospiraceae*, *Porphyromonadaceae*, and *Ruminococcaceae* [90]. Members of these families may participate in nutrient cycling and the degradation of organic matter, thereby supporting uterine homeostasis.

Culture-based and amplicon sequencing studies consistently identify *Bacillus*, *Fusobacterium*, and *Porphyromonas* among the most prevalent genera within the bovine uterus [91]. Similarly, Bacillus and Enterococcus were reported as dominant genera in the uterine microbiota of Canadian Holstein-Friesian cows irrespective of estrous cycle stage, whereas *Lachnospiraceae*, *Oscillospiraceae*, and *Streptococcus* were present in lower abundance [92]. Interestingly, *F. necrophorum*, *Porphyromonas levii*, and *T. pyogenes* have been localized within the endometrium and caruncular stroma, without a clear association with inflammation, suggesting that their mere presence may not be sufficient to induce disease [90]. Hummel et al. [93] further reported a negative correlation between bacterial abundance in the uterus and bloodstream, suggesting that reductions in circulating bacterial load may correspond to increased uterine bacterial colonization.

## 12. Postpartum Microbial Dynamics

During the postpartum period, dairy cows frequently experience negative energy balance (NEB) because energy demands for lactation exceed dietary intake. Cows experiencing NEB exhibit uterine microbiota dominated by Bacteroidetes and Fusobacteria, whereas healthier microbial communities are characterized by greater relative abundances of Proteobacteria and Firmicutes [94]. As NEB progresses, the microbial profile may further shift toward Actinobacteria, Cyanobacteria, and Proteobacteria [94].

Seasonal variation also influences the uterine microbiota. *Enterobacteriaceae*, *Lactobacillaceae*, *Moraxellaceae*, *Ruminococcaceae*, and *Staphylococcaceae* are more prevalent during summer, whereas *Clostridiaceae*, *Bacteroidaceae*, *Lachnospiraceae*, *Moraxellaceae*, and *Ruminococcaceae* dominate during winter [95]. These differences may reflect seasonal variation in airborne dust and bedding microbiota. In summer, the use of misting fans and cooling systems may further modify interactions among environmental microorganisms, bedding, and the uterine microbiota.

During the postpartum period, the uterus undergoes extensive remodeling, tissue repair, and microbial clearance [3]. Bacterial contamination of the uterine lumen is common after calving and, in healthy cows, is usually resolved without long-term consequences [3,96,97]. However, failure to eliminate bacterial contamination may result in postpartum uterine diseases such as metritis and endometritis [4,96]. Recent microbiome studies suggest that disease progression depends not only on the presence of pathogens but also on alterations in microbial community structure and diversity [98]. Following parturition, physical barriers are disrupted, enabling bacteria originating from the vagina, gastrointestinal tract, and surrounding environment to colonize the uterus [99,100]. Although bacterial colonization occurs almost universally during the first two postpartum weeks, the resulting microbial dynamics are influenced by host immunity, metabolic status, and endocrine factors. Cows that develop metritis or endometritis typically exhibit increased abundance of pathogenic or opportunistic bacteria accompanied by reduced microbial diversity and depletion of beneficial commensal populations [38].

## 13. Dysbiosis and Uterine Disease

Historically, investigations of the uterine microbiota relied primarily on aerobic and anaerobic culture techniques. These studies identified several bacterial species associated with uterine disease. In cows with endometritis, commonly isolated bacteria include *T. pyogenes*, *F. necrophorum*, *E. coli*, *Prevotella* spp., *Bacteroides* spp., and hemolytic *Streptococci* [101,102]. Metritis is associated with similar bacterial populations, including *E. coli*, *T. pyogenes*, *F. necrophorum*, *Streptococcus* spp., *Staphylococcus* spp., and *Pseudomonas* spp. [20]. However, culture-based methods underestimate microbial diversity because many organisms are difficult or impossible to culture under standard laboratory conditions [103]. Consequently, molecular approaches such as next-generation sequencing have transformed the understanding of the bovine uterine microbiota.

Studies using 16S rRNA gene sequencing demonstrate that the uterine microbiota of both healthy and diseased cows is dominated by bacteria belonging to the phyla Firmicutes, Bacteroidetes, Fusobacteria, Proteobacteria, and Tenericutes [86,98,103]. In healthy cows, *Lactobacillus* spp., *Propionibacterium* spp., and other members of Firmicutes and Actinobacteria are frequently observed and are thought to support reproductive health by maintaining low pH, inhibiting pathogen colonization, and modulating local immune responses [14,20,104]. In contrast, cows with uterine disease often exhibit increased abundance of *Fusobacterium* spp., *Bacteroides* spp., *Prevotella* spp., *Ureaplasma* spp., and *Trueperella* spp. [14,20,104,105]. These organisms are associated with inflammation, tissue damage, and impaired reproductive performance. For example, *F. necrophorum* produces leukotoxin (ltxA), which suppresses host immune cell activity, whereas *T. pyogenes* produces pyolysin, a cytolytic toxin that damages endometrial cells [106].

Longitudinal studies indicate that uterine microbial communities change dynamically throughout the postpartum period [107]. During the first postpartum week, healthy and diseased cows may harbor similar bacterial populations, including Fusobacterium, Bacteroidetes, and Firmicutes. However, by approximately 30 days postpartum, healthy cows generally exhibit increased abundance of Firmicutes and Proteobacteria, whereas diseased cows frequently demonstrate persistent overgrowth of Tenericutes, Synergistetes, and other opportunistic pathogens [104,105,107]. These findings suggest that persistence and dysbiosis, rather than initial bacterial exposure alone, are critical determinants of disease development.

### 13.1. Vaginal–Uterine Microbial Interactions

The vagina serves as an important microbial reservoir for bacteria ascending into the uterus during and after calving [108,109]. Consequently, vaginal microbiota composition may substantially influence uterine health. Elevated vaginal populations of *E. coli* or *T. pyogenes* during the periparturient period are associated with increased risk of postpartum uterine disease [110]. Therefore, strategies aimed at modulating the vaginal microbiota, including improved hygiene, probiotic administration, or prepartum interventions, may offer preventive benefits.

The postpartum uterus and vagina form a connected microbial ecosystem, with substantial microbial exchange occurring during calving. Vaginal colonization by *E. coli* or *T. pyogenes* can therefore act as a reservoir for uterine infection [88]. Interventions targeting the vaginal microbiota before or immediately after calving may help reduce the risk of postpartum uterine disease.

Metatranscriptomic approaches provide additional insight into microbial function by evaluating bacterial gene expression profiles rather than simply identifying microbial presence. Studies examining uterine microbial transcripts have demonstrated increased expression of genes encoding toxins, adhesins, proteases, and siderophores in cows with uterine disease [16,111,112,113]. Functional characterization of these virulence-associated pathways may facilitate development of novel therapeutic targets and precision-based interventions.

### 13.2. From Bacterial Presence to Functional Dysbiosis

Collectively, these studies suggest that postpartum uterine disease cannot be explained solely by the detection of individual bacterial species. Rather than representing infection by a single pathogen, disease appears to emerge from ecological disruption within the postpartum uterine microbiome. Several studies demonstrate that potentially pathogenic organisms may also be detected in clinically healthy cows, indicating that microbial abundance alone is insufficient to predict disease. Instead, alterations in microbial diversity, community structure, temporal succession, and bacterial functional activity appear to better distinguish healthy uterine recovery from persistent inflammation. Likewise, metatranscriptomic investigations indicate that changes in virulence gene expression may be more informative than taxonomic composition alone. These observations support a conceptual shift from pathogen-centered models toward ecological models of postpartum uterine disease, in which microbial dysbiosis, impaired immune regulation, and delayed tissue repair interact to determine disease progression.

### 13.3. Current Knowledge Gaps and Future Research Needs

Despite rapid advances in culture-independent sequencing technologies, several important questions remain unresolved. First, considerable variability exists among published studies in sampling strategy, postpartum sampling time, sequencing platforms, DNA extraction methods, and bioinformatic pipelines, making direct comparisons across studies difficult and contributing to inconsistent findings. Second, although dysbiosis is consistently associated with postpartum uterine disease, it remains unclear whether microbial dysbiosis is a primary driver of inflammation or a secondary consequence of an already compromised uterine environment. Third, many investigations focus primarily on taxonomic composition without simultaneously evaluating microbial function, host immune responses, endocrine regulation, metabolic status, and reproductive outcomes. Consequently, important mechanistic links between microbial community dynamics and disease progression remain poorly defined. Future research should prioritize standardized longitudinal studies that integrate metagenomics, metatranscriptomics, metabolomics, host immunophenotyping, and reproductive phenotyping to establish causal relationships between microbial ecology and postpartum uterine disease and to identify clinically relevant biomarkers and therapeutic targets.

Collectively, available studies indicate that postpartum uterine disease is determined less by the presence of individual bacterial species than by failure of microbial succession toward a stable, diverse microbial community accompanied by restoration of immune homeostasis. Thus, dysbiosis should be viewed as a disruption of ecosystem function rather than merely replacement of one pathogen by another.

## 14. Bacteria Associated with Postpartum Uterine Disease

The exact relationship between specific pathogens and postpartum uterine disease remains incompletely understood; however, certain bacterial species are consistently associated with either healthy or diseased uterine conditions. Most investigations of postpartum uterine infections in dairy cows have relied on traditional culture-based techniques to identify bacterial populations [114]. More recently, pyrosequencing and next-generation sequencing studies have provided additional insight into the postpartum uterine microbiota.

Metagenomic analyses have identified bacterial taxa associated with both healthy and diseased uterine environments. *Lactobacillus* spp. belonging to the phylum Firmicutes and *Propionibacterium* spp. from the phylum Actinobacteria are frequently associated with favorable reproductive health [115,116]. Additionally, Proteobacteria and Tenericutes have been identified in the uterine microbiota of clinically healthy cows. Studies utilizing next-generation sequencing and denaturing gradient gel electrophoresis have consistently identified Bacteroidetes, Fusobacteria, Firmicutes, Proteobacteria, and Tenericutes in the uterine microbiota irrespective of disease status [115,116,117].

### 14.1. Bacteria Identified Using Traditional Culture Methods

#### 14.1.1. Endometritis

*T. pyogenes*, *Prevotella melaninogenicus*, *E. coli*, *F. necrophorum*, *Prevotella* spp., *hemolytic streptococci*, *Bacteroides* spp., *Pasteurella* spp., and *Proteus* spp.

#### 14.1.2. Metritis

*E. coli*, *T. pyogenes*, *F. necrophorum*, *Streptococcus* spp., *Staphylococcus* spp., *Pseudomonas* spp., *Prevotella* spp., and *Bacteroides* spp.

Several studies have investigated temporal changes in uterine bacterial populations during the postpartum period. Fusobacterium, Bacteroidetes, and Tenericutes were detected in healthy cows during the first week postpartum using next-generation sequencing approaches. Similarly, Bacteroidetes, Firmicutes, and Fusobacterium were predominant in cows with metritis during this period. Approximately one month postpartum, Firmicutes and Proteobacteria dominated the uterine microbiota of healthy cows, whereas Tenericutes, Synergistetes, and Proteobacteria were more abundant in cows with endometritis [14,115,116,117]. A terminal restriction fragment length polymorphism (T-RFLP) study demonstrated that *F. necrophorum*, *Pseudomonas* spp., *Acinetobacter* spp., and *Bacteroides* spp., Sphingobacterium, and Prevotellaceae remained prevalent both one week and one month postpartum, regardless of uterine health status [118].

### 14.2. Bacteria Identified Using Next-Generation Sequencing

#### 14.2.1. Endometritis

Bacteroidetes, Fusobacteria, Firmicutes, Proteobacteria, *Bacteroides* spp., *Ureaplasma* spp., *Fusobacterium* spp., *Peptostreptococcus* spp., *Sneathia* spp., *Prevotella* spp., and *Trueperella* spp.

#### 14.2.2. Metritis

Fusobacteria, Bacteroidetes, Proteobacteria, Firmicutes, *Bacteroides* spp., and *Ureaplasma* spp.

### 14.3. Metatranscriptomics and Virulence Factors

Metatranscriptomics involves analyzing the complete pool of microbial transcripts within a bacterial community and provides insight into the functional activity of the microbiota [119]. By applying next-generation sequencing to RNA transcripts, researchers can identify genes actively expressed within the postpartum uterine microbiota. Comparative analyses between healthy cows and cows with postpartum uterine disease may reveal genes involved in the establishment, persistence, and virulence of infection [120]. Although metatranscriptomic studies have been widely applied to environmental and human-associated microbiomes, investigations of the bovine uterine metatranscriptome remain limited.

## 15. Potential Virulence Factors of Uterine Pathogens

Virulence refers to a microorganism’s capacity to cause damage or disease within a host, whereas pathogenicity encompasses virulence along with factors influencing disease transmission and progression. Pathogenesis describes the mechanisms by which pathogens induce disease, and virulence factors are microbial genes or products that contribute to pathogenic potential.

Several virulence factors associated with major uterine pathogens, including *E. coli*, *T. pyogenes*, and *F. necrophorum*, have been identified using quantitative PCR approaches.

### 15.1. Key Virulence Factors of Uterine Pathogens

*E. coli*: *fimH*, *astA*, *cdt*, *kpsMII*, *ibeA*, *hlyA*, *fyuA*

*T. pyogenes*: *plo*, *cbpA*, *fimA*, *fimG*, *nanH*, *nanP*

*F. necrophorum*: *lktA*, *lktB*, *lktC*, *hly*, hemagglutinin, endotoxin, adhesins, dermonecrotic toxin, platelet aggregation factor, and proteases

### 15.2. Virulence and Pathogenesis

#### 15.2.1. *Escherichia coli*

*E. coli* is a Gram-negative, facultative anaerobic bacterium belonging to the family Enterobacteriaceae within the phylum Proteobacteria. Although many strains exist as commensal organisms within the gastrointestinal tract, pathogenic variants are associated with numerous diseases, including diarrhea, urinary tract infections, and postpartum uterine disease in dairy cattle. Among its virulence determinants, the type 1 pilus encoded by the *fimH* gene plays a critical role in bacterial adhesion, colonization, and invasion of the uterine epithelium. Studies have demonstrated that uterine pathogenic *E. coli* (UPEC) strains facilitate colonization by other opportunistic pathogens, including *F. necrophorum* and *T. pyogenes*, thereby contributing to progression of postpartum uterine disease [111]. Furthermore, expression of *fimH* is strongly associated with uterine pathogenicity and inflammatory responses within the postpartum uterus.

*E. coli* is among the earliest bacterial species to colonize the uterus during the immediate postpartum period. Excessive proliferation of pathogenic strains is frequently associated with acute metritis and clinical endometritis. UPEC strains commonly express multiple virulence factors that enhance persistence and pathogenicity within the uterine environment. These include *FimH* adhesins, which facilitate attachment to endometrial epithelial cells; LPS, a potent endotoxin that activates inflammatory pathways via TLR4; and iron-acquisition systems, such as siderophores, that enhance bacterial survival under iron-limited conditions characteristic of the postpartum uterus. Activation of innate immune pathways by *E. coli* LPS stimulates neutrophil infiltration and promotes production of pro-inflammatory cytokines, including IL-1β and TNF-α. Although these immune responses are essential for pathogen clearance, excessive or prolonged inflammation may impair endometrial repair, disrupt ovarian function, delay uterine involution, and ultimately reduce fertility in affected cows [121].

#### 15.2.2. *Trueperella pyogenes*

*T. pyogenes* is a Gram-positive, facultative anaerobic opportunistic pathogen associated with mastitis, pneumonia, and uterine infections. Important virulence factors include pyolysin, encoded by the plo gene, and neuraminidase, encoded by *nanH*, which contributes to bacterial adhesion by cleaving host sialic acids [122].

*T. pyogenes* contributes to uterine pathology through:

- Pyolysin-mediated cytotoxicity and immune suppression;
- Production of proteases and exoenzymes facilitating tissue degradation;
- Synergistic interactions with anaerobic bacteria such as *F. necrophorum.*

This organism efficiently exploits impaired uterine immune defenses and proliferates within necrotic tissue, contributing to purulent discharge and endometrial damage.

Additional virulence factors include fimbriae (*Fim*), neuraminidases (*NanH* and *NanP*), and collagen-binding protein (CbpA) [123,124]. Pyolysin remains the principal virulence determinant [125,126], mediating cell lysis and hemolysis [127,128]. Other virulence-associated genes contribute to bacterial adherence, colonization, and disease progression [126,129].

Previous studies demonstrated differences in the expression of multiple virulence genes among *T. pyogenes* isolates obtained from cows with varying uterine health status [130], underscoring the importance of virulence regulation in disease pathogenesis [126,131]. Liu et al. [132] further reported differences in cytolytic activity among *T. pyogenes* isolates recovered from uterine lavage fluid. Collectively, these findings suggest that additional unidentified bacterial factors may contribute to pathogenicity.

Combined transcriptomic and proteomic analyses have enhanced understanding of *T. pyogenes* pathogenic mechanisms [133]. Strains TP1804 and TP1808, isolated from cows with endometritis, expressed *plo, nanP, cbpA, fimA, fimC*, and *fimE* [132]. Although both strains demonstrated similar growth kinetics, they exhibited different levels of cytotoxicity, indicating that regulation of virulence-associated genes and proteins may substantially influence pathogenic potential. Investigating these regulatory mechanisms may facilitate the identification of novel biomarkers and improve diagnostic and therapeutic strategies.

#### 15.2.3. *Fusobacterium necrophorum*

*F. necrophorum* is a Gram-negative, obligate anaerobic bacterium associated with several bovine diseases, including postpartum uterine infections. Its principal virulence factor is leukotoxin encoded by *lktA*, which contributes to immune evasion by damaging host leukocytes [134,135]. Additional virulence factors include hemolysins, adhesins, and proteases that facilitate tissue colonization and destruction.

Understanding the uterine microbiota and its contribution to postpartum uterine disease may support the development of novel preventive and therapeutic strategies while also improving understanding of antimicrobial resistance mechanisms.

**Figure 1:**
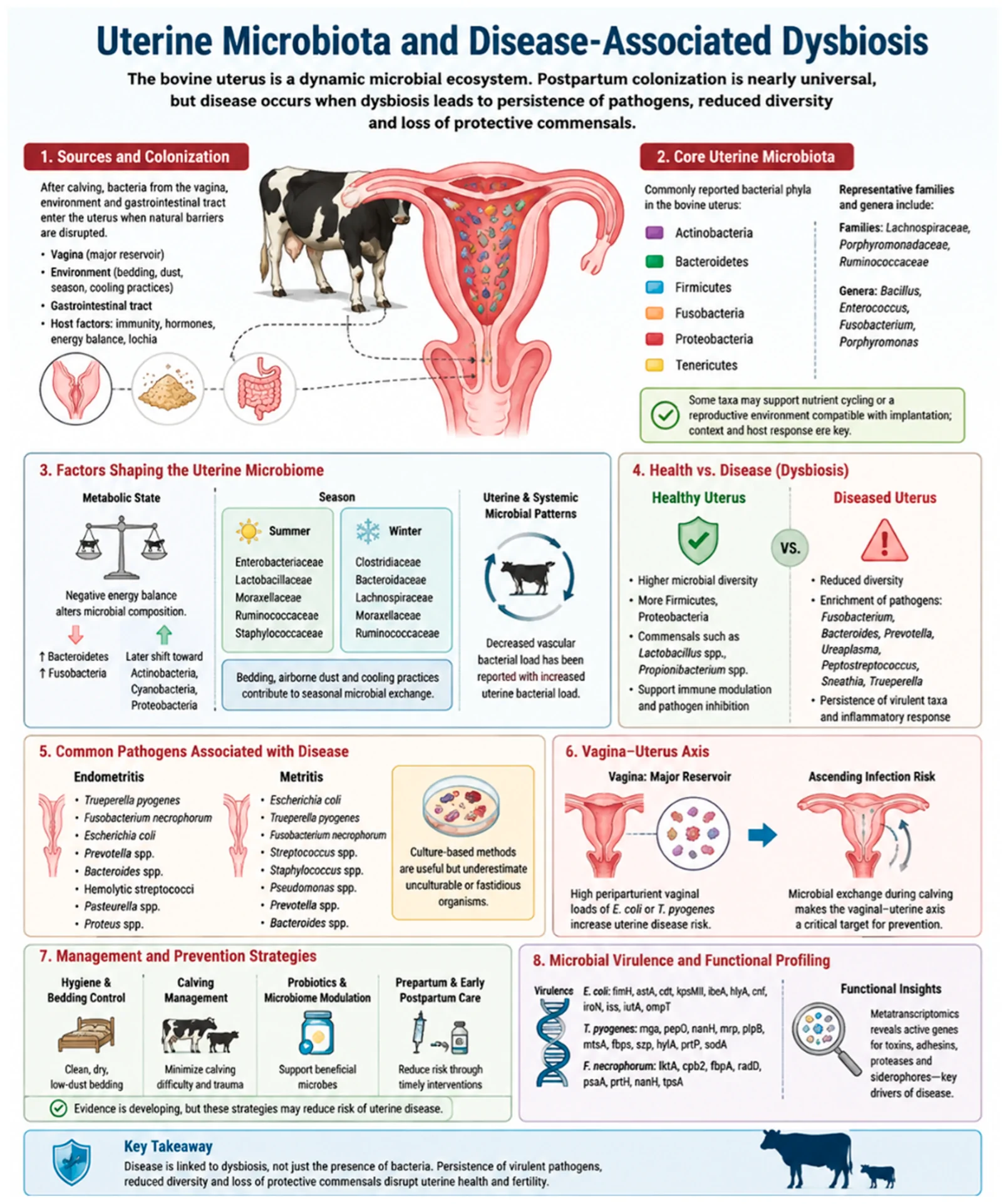
Schematic overview of uterine microbiota, dysbiosis, and microbial virulence associated with postpartum uterine disease in dairy cattle.

Figure 1 illustrates the dynamic postpartum bovine uterine microbiome and the transition from physiological bacterial colonization to disease-associated dysbiosis. Following calving, bacteria originating from the vagina, gastrointestinal tract, and environment colonize the uterus when natural physical barriers are disrupted. Core uterine microbial phyla include Actinobacteria, Bacteroidetes, Firmicutes, Fusobacteria, Proteobacteria, and Tenericutes. Metabolic state, seasonal factors, environmental exposure, and microbial exchange along the vagina–uterus axis shape uterine microbial composition. Healthy uteri are characterized by greater microbial diversity and enrichment of potentially protective commensals, whereas diseased uteri exhibit reduced diversity and persistence of pathogenic taxa, including *Fusobacterium* spp., *Bacteroides* spp., *Prevotella* spp., *Trueperella* spp., and *Ureaplasma* spp. The figure further summarizes major pathogens associated with metritis and endometritis, including *E. coli*, *T. pyogenes*, and *F. necrophorum*, together with key virulence factors involved in adhesion, immune evasion, cytotoxicity, and tissue damage. Potential prevention and management strategies, including hygiene, calving management, probiotics, and microbiome modulation, are also highlighted. The figure is adapted from concepts discussed in the review of bovine uterine microbiota and disease-associated dysbiosis.

## 16. Dairy Heifers: A Stable and Low-Risk Microbiota

In dairy heifers and primiparous cows, the uterine microbiota is generally more stable and less diverse, with lower pathogen abundance than in multiparous cows. Common commensal bacterial genera identified within the reproductive tract include *Lactobacillus*, *Trueperella*, *Ruminococcus*, and *Bacteroides* [136]. Because primiparous animals possess an intact cervix and have not previously undergone repeated calving-associated trauma, ascending bacterial infections and excessive uterine contamination are generally less common. Furthermore, primiparous cows are typically exposed to lower cumulative metabolic and reproductive stress than older multiparous cows, which may contribute to more effective immune function and improved uterine recovery during the postpartum period. As a result, primiparous cows often exhibit lower incidence and severity of postpartum uterine diseases, including metritis and endometritis.

In contrast, multiparous cows are more susceptible to uterine dysbiosis and postpartum uterine disease because of repeated exposure to calving-related tissue damage, metabolic stress, and high milk production demands. These cows frequently experience more pronounced negative energy balance, greater fat mobilization, and impaired immune competence during early lactation, all of which compromise uterine defense mechanisms and pathogen clearance. Additionally, repeated parturitions may alter the reproductive tract microenvironment facilitating colonization by pathogenic microorganisms such as *E. coli*, *F. necrophorum*, and *T. pyogenes*. Multiparous cows also tend to exhibit delayed uterine involution, increased inflammatory responses, and higher prevalence of retained placenta, dystocia, and metabolic disorders, further increasing susceptibility to postpartum infections. Studies have shown that the uterine microbiota of multiparous cows is often characterized by greater microbial diversity and higher abundance of opportunistic pathogens associated with persistent inflammation and reduced fertility.

Despite the lower risk of uterine disease in heifers and primiparous cows, maintaining of proper hygiene, nutritional management, and immune competence remains essential as animals approach parturition. Collectively, these findings indicate that parity significantly influences uterine microbial composition, immune regulation, and postpartum reproductive health, highlighting important physiological and microbiological differences between primiparous and multiparous dairy cows.

## 17. Rumen Microbiota, Systemic Metabolism, and Uterine Health

Although the rumen microbiota is anatomically distinct from the reproductive tract, increasing evidence suggests that ruminal microbial activity may indirectly influence postpartum uterine health by affecting systemic metabolism, immune competence, inflammation, and transition cow physiology. The rumen microbiota plays a central role in nutrient digestion, volatile fatty acid production, energy balance, and regulation of host metabolic homeostasis. Consequently, disturbances in ruminal microbial composition during the transition period may contribute to metabolic disorders that predispose dairy cows to postpartum uterine disease [64,66,82,83,84,85].

High-producing dairy cows commonly experience NEB during early lactation because energy demands for milk synthesis exceed dietary intake. Alterations in ruminal fermentation and microbial composition during this period can influence feed efficiency, ruminal pH, nutrient absorption, and production of metabolites involved in immune and endocrine regulation. Dysregulation of ruminal microbial ecology has been associated with ketosis, subacute ruminal acidosis, excessive fat mobilization, systemic inflammation, and impaired neutrophil function, all of which are recognized risk factors for metritis and endometritis [53,54,64,66].

In addition to metabolic effects, ruminal microbial products may influence systemic immune responses through the gut–immune axis. Lipopolysaccharides derived from Gram-negative ruminal bacteria can translocate into circulation under conditions of impaired ruminal barrier integrity, contributing to systemic inflammation and altered cytokine signaling [21,22,48,49,50,51,52,53,54,55]. Elevated circulating inflammatory mediators may impair uterine immune defenses, delay uterine involution, and compromise endometrial repair during the postpartum period. Furthermore, nutritional deficiencies associated with altered rumen function may reduce antioxidant capacity and impair leukocyte activity, thereby decreasing the efficiency of uterine pathogen clearance [64,65].

Emerging evidence also suggests that interactions among the gastrointestinal, vaginal, and uterine microbiota may contribute to microbial exchange and reproductive health [88,99,108,109,110]. Because the postpartum uterus is highly susceptible to environmental and ascending bacterial contamination, systemic metabolic stress originating from gastrointestinal dysbiosis may indirectly influence the composition and resilience of the uterine microbial ecosystem. Consequently, maintaining of ruminal health during the transition period may represent an important component of integrated strategies to improve immune competence, reduce postpartum inflammation, and support uterine recovery.

Although direct mechanistic links between the rumen microbiota and postpartum uterine microbiota remain incompletely understood, the available evidence supports the concept that ruminal microbial homeostasis contributes indirectly to reproductive health through coordinated effects on metabolism, immunity, inflammation, and transition cow adaptation [78,82,83,84,85]. Future multi-omics studies integrating ruminal, vaginal, and uterine microbiomes, along with metabolic and immunological profiling, may provide deeper insight into these interconnected host–microbe interactions and their implications for postpartum reproductive efficiency in dairy cattle.

## 18. Integrated Model and Translational Priorities

The postpartum uterus should be viewed as a dynamic ecological and physiological interface rather than a passive site of bacterial contamination. Development of postpartum uterine disease results from complex interactions among microbial colonization, host immune competence, endocrine recovery, metabolic adaptation, tissue remodeling, and environmental management during the transition period [13,14,15,16,17,21,22,23,37]. Although bacterial contamination of the uterus after calving is nearly universal, disease occurs when microbial burden and virulence exceed the capacity of host defense and uterine repair mechanisms [3,4,5,6,7]. Consequently, postpartum uterine disease is best understood as a multifactorial dysbiosis-driven disorder arising from disruption of host–microbe homeostasis rather than from the presence of a single pathogen alone.

Parturition disrupts normal physical barriers of the reproductive tract and exposes the uterus to microorganisms originating from the vagina, gastrointestinal tract, bedding, and surrounding environment [99,100,108,109]. Early colonization by facultative pathogens, particularly *E. coli*, promotes inflammation, oxygen depletion, and tissue damage that favor subsequent proliferation of anaerobic organisms such as *F. necrophorum*, *T. pyogenes*, and *Prevotella* spp. [13,14,15,16,17,38,39,40,98,101,102,103,104,105]. These microorganisms interact synergistically through the production of endotoxins, leukotoxins, pyolysin, adhesins, proteases, and other virulence-associated factors that enhance bacterial persistence, suppress host immunity, and exacerbate endometrial injury [106,111,112,113,114,115,116,117,118,119,120,121,122,123,124,125,126,127,128,129,130,131,132,133,134,135]. At the same time, postpartum cows experience substantial metabolic and endocrine stress associated with lactation, negative energy balance, hypocalcemia, and altered progesterone and estradiol signaling, all of which impair neutrophil function, cytokine regulation, and uterine clearance capacity [23,35,36,53,54,64,65,66]. The resulting inflammatory microenvironment contributes to delayed uterine involution, persistent dysbiosis, oxidative stress, impaired ovarian cyclicity, and reduced fertility [34,41,42,43,44,45].

This integrated ecological model helps explain why antimicrobial treatment alone often fails to fully restore reproductive performance. By the time clinical disease becomes detectable, substantial endocrine disruption, immune dysregulation, microbial succession, and tissue injury may already be established [41,42,43,44,45,55]. Furthermore, indiscriminate antimicrobial use may contribute to antimicrobial resistance, disruption of beneficial microbial communities, and incomplete restoration of uterine homeostasis. Consequently, future management strategies should move beyond pathogen elimination alone and instead focus on restoring physiological balance within the postpartum reproductive tract [16,67,111,112,113].

From a translational perspective, precision-based reproductive management strategies are needed to improve early disease detection, optimize treatment selection, and reduce unnecessary antimicrobial exposure. Integration of clinical assessment with cytology, ultrasonography, inflammatory biomarkers, metabolic indicators, and microbiome profiling may facilitate identification of cows at highest risk for persistent uterine inflammation before severe reproductive impairment occurs [11,12,55,56,57,58,59,60,61,62,63]. Candidate biomarkers include acute-phase proteins, cytokine profiles, polymorphonuclear neutrophil percentages, metabolic markers of negative energy balance, microbial virulence transcripts, and host transcriptomic signatures associated with impaired uterine recovery [47,48,61,62,63,111,112,113,114,115,116,117,118,119,120]. Combining host-response biomarkers with functional microbial profiling may provide more accurate prediction of disease progression than reliance on bacterial culture or clinical signs alone [18,19,20,119,120].

### 18.1. Established Evidence

Culture-independent sequencing technologies, particularly 16S rRNA gene sequencing, have substantially advanced understanding of the postpartum uterine microbiota by revealing microbial diversity that cannot be captured using conventional culture-based techniques. Collectively, these studies consistently demonstrate that postpartum uterine disease is associated with alterations in microbial community structure, reduced microbial diversity, and disruption of normal microbial succession rather than the simple presence or absence of individual bacterial pathogens. Integration of microbiological findings with host immune responses has further established that successful postpartum uterine recovery depends on coordinated microbial clearance, inflammation resolution, and restoration of uterine homeostasis. These observations now represent well-supported concepts within the field and provide the biological foundation for current diagnostic and preventive strategies.

### 18.2. Emerging Approaches

Although recent technological advances offer considerable promise, several emerging approaches remain at an early stage of investigation and require prospective validation before routine clinical implementation. Whole-metagenome sequencing, metatranscriptomics, metabolomics, and other multi-omics platforms have the potential to identify functional microbial pathways, characterize host–microbe interactions, and discover biomarkers predictive of disease progression. Likewise, microbiome-guided therapeutic strategies, probiotic or microbiota-modulating interventions, and artificial intelligence-based predictive models may ultimately facilitate precision diagnosis and individualized herd management. However, these approaches are currently limited by methodological variability, differences in sampling protocols, lack of standardized analytical pipelines, relatively small longitudinal datasets, and insufficient validation under commercial dairy production conditions. Future research integrating microbial function, host immunity, endocrine regulation, metabolic status, and reproductive outcomes will be essential before these technologies can be translated into routine veterinary practice.

Future therapeutic approaches should emphasize antimicrobial stewardship and the development of targeted interventions that preserve microbial homeostasis while limiting the emergence of antimicrobial resistance. Selective treatment protocols, pathogen-directed therapies, immunomodulatory approaches, anti-inflammatory strategies that do not impair pathogen clearance, probiotics, microbiome-restorative therapies, and nutritional support during the transition period all represent promising alternatives to routine broad-spectrum antimicrobial use [67,88,108,109,110,136]. Prepartum management also remains critically important. Optimizing transition nutrition, minimizing excessive body condition loss, improving calving hygiene, reducing dystocia, and rapidly managing retained fetal membranes may substantially reduce postpartum disease risk by improving immune competence and limiting early microbial overgrowth [56,57,58,63,64,65,66,67,68,69,137,138,139].

Advances in next-generation sequencing, metagenomics, metatranscriptomics, metabolomics, and systems biology are expected to further refine understanding of host–microbe interactions within the postpartum uterus [18,19,20,111,112,113,114,115,116,117,118,119,120]. Longitudinal multi-omics studies integrating microbial, endocrine, metabolic, and immunological data will likely improve identification of causal mechanisms underlying dysbiosis and reproductive failure. Ultimately, integrating microbiology, immunology, reproductive physiology, precision diagnostics, and herd-level management strategies will be essential for improving fertility, animal welfare, antimicrobial stewardship, and the long-term sustainability of modern dairy production systems [67,111,112,113,114,115,116,117,118,119,120].

**Figure 2:**
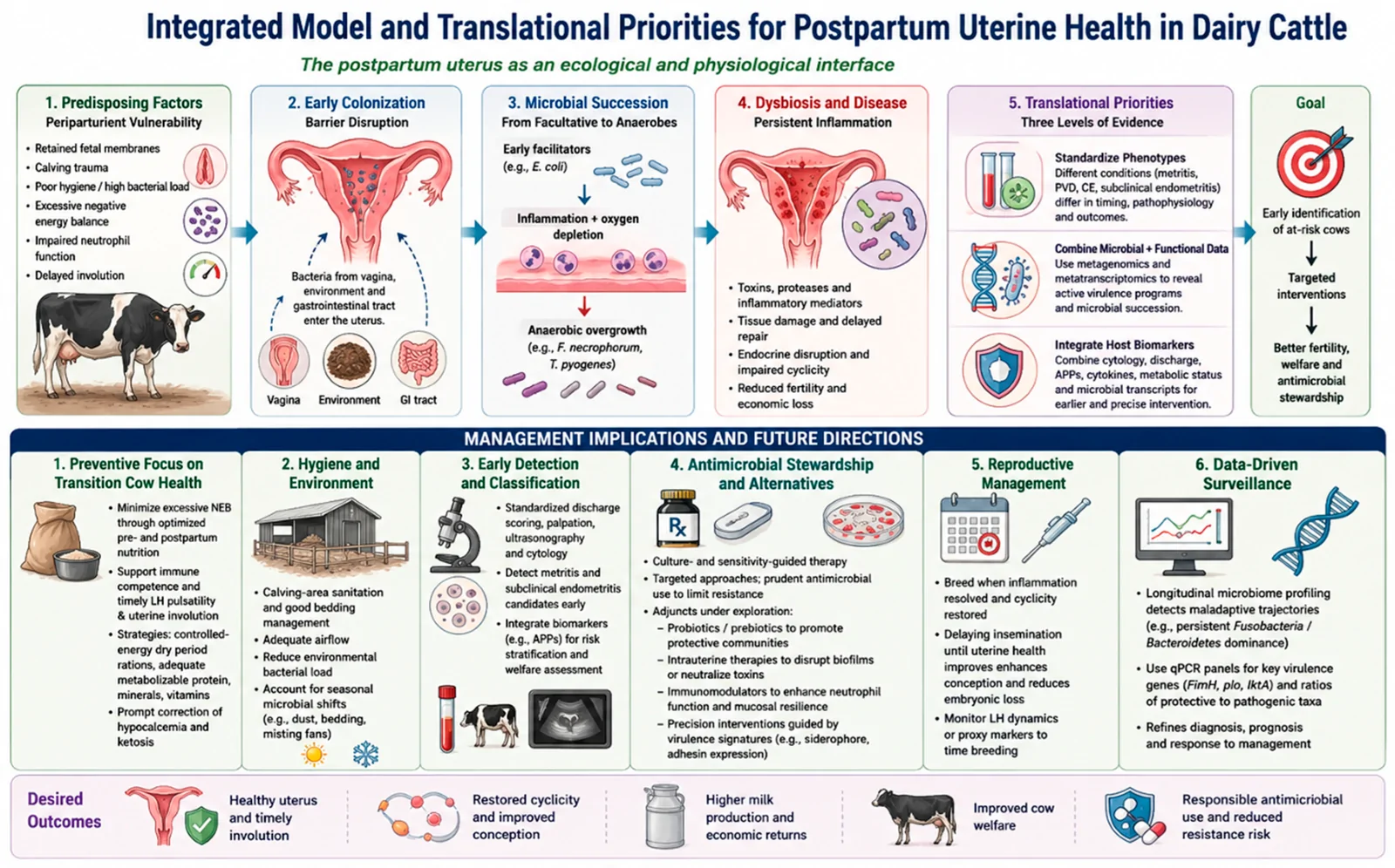
Integrated model and translational priorities for postpartum uterine health in dairy cattle. (**Top**) Disease emerges when microbial pressure exceeds host clearance capacity during a period of vulnerability, leading to microbial succession, toxin production, and persistent inflammation that disrupt fertility. (**Bottom**) Key management implications and future directions emphasize prevention, early detection, precise therapy, and data-driven decision making to improve reproductive performance and sustainability.

Figure 2 summarizes the ecological and physiological framework underlying postpartum uterine disease development and highlights major translational and management priorities. Predisposing factors, including retained fetal membranes, calving trauma, poor hygiene, excessive negative energy balance, impaired neutrophil function, and delayed uterine involution, increase host susceptibility during the transition period. Following disruption of physical barriers after calving, bacteria originating from the vagina, gastrointestinal tract, and environment colonize the uterus. Early facultative organisms, particularly *E. coli*, promote inflammation and oxygen depletion, thereby facilitating microbial succession toward anaerobic pathogens such as *F. necrophorum* and *T. pyogenes*. Persistent dysbiosis results in toxin production, tissue damage, endocrine disruption, delayed uterine repair, impaired cyclicity, and reduced fertility. The figure further outlines translational priorities, including standardized phenotypic definitions, integration of microbiome composition with functional and virulence profiling, and incorporation of host biomarkers for precision diagnostics. Management implications emphasize transition cow nutrition, hygiene, and environmental control; early detection; antimicrobial stewardship; reproductive management; and longitudinal microbiome surveillance. Collectively, these integrated strategies aim to improve uterine recovery, reproductive efficiency, animal welfare, and responsible antimicrobial use in dairy herds.

## 19. Current Limitations and Outstanding Questions

Despite major advances in sequencing technologies and reproductive immunology, several important limitations continue to challenge the interpretation of postpartum uterine microbiome studies. First, the bovine uterus represents a low-biomass microbial environment, making studies particularly vulnerable to environmental contamination, reagent-derived bacterial DNA, and sampling-associated bias. Consequently, distinguishing true resident microbial populations from transient contamination remains difficult. Second, substantial variation exists among studies in sampling methodology, sequencing platforms, bioinformatic pipelines, and disease definitions, limiting comparability across datasets. Third, most available microbiome studies remain observational and therefore establish association rather than causation between microbial populations and disease progression.

Future studies should integrate metagenomics, metatranscriptomics, metabolomics, and host immune profiling to better characterize functional host–microbe interactions during postpartum uterine recovery. Longitudinal studies evaluating microbial succession together with endocrine, metabolic, and immunological parameters will be essential for distinguishing physiological postpartum adaptation from pathological dysbiosis. Additional research is also needed to identify reproducible microbial biomarkers, validate precision diagnostic tools, evaluate microbiome-restorative therapies, and improve antimicrobial stewardship in dairy production systems.

## 20. Conclusions

Postpartum uterine diseases remain among the most significant reproductive disorders affecting dairy cattle because of their profound effects on fertility, productivity, animal welfare, and farm profitability. Contemporary evidence indicates that disease pathogenesis results from complex interactions among microbial colonization, virulence regulation, immune competence, endocrine recovery, metabolic status, and environmental management during the postpartum transition period. Although traditional investigations emphasized isolation of individual pathogens, modern sequencing technologies increasingly support a dysbiosis-based model in which microbial community structure, functional activity, and host–microbe interactions collectively determine disease progression.

Advances in metagenomics, metatranscriptomics, and systems biology approaches are expanding understanding of postpartum uterine ecology and may facilitate development of precision diagnostics, microbiome-informed therapeutics, and targeted prevention strategies. Future progress will depend on integrating microbial profiling with host immune, endocrine, and metabolic biomarkers to identify cows at greatest risk for persistent inflammation and reproductive failure. Improved transition management, antimicrobial stewardship, and precision herd-health strategies will be essential for optimizing reproductive performance while reducing unnecessary antimicrobial use. Collectively, continued integration of microbiology, reproductive physiology, immunology, and translational dairy management will be critical for improving uterine health and long-term sustainability of modern dairy systems.

Collectively, current evidence supports a transition from viewing postpartum uterine disease as a simple bacterial infection toward understanding it as a multifactorial ecological disorder resulting from interactions among microbial succession, immune competence, endocrine adaptation, metabolic status, and tissue repair. Continued integration of microbiology, immunology, reproductive physiology, and systems biology will be essential for developing precision diagnostics and targeted interventions that improve fertility while reducing antimicrobial use in dairy production systems.

## Data Availability

No new data were created or analyzed in this study. Data sharing is not applicable to this article.
